# The Harder the Heart, the Harder It Breaks: A Case of Complete Atrioventricular Block Secondary to Tertiary Hyperparathyroidism

**DOI:** 10.7759/cureus.13276

**Published:** 2021-02-11

**Authors:** Ramanakumar Anam, Mauricio Tellez, Matthew Lozier, Ameen Waheed, Jose E Collado

**Affiliations:** 1 Internal Medicine, University of Miami at Holy Cross Hospital, Fort Lauderdale, USA; 2 Cardiology, Mount Sinai Medical Center, Miami, USA; 3 Nephrology, University of Miami at Holy Cross Hospital, Fort Lauderdale, USA; 4 Cardiology, University of Miami at Holy Cross Hospital, Fort Lauderdale, USA; 5 Cardiology, Jim Moran Heart & Vascular Center at Holy Cross Hospital, Fort Lauderdale, USA

**Keywords:** mitral annular calcification, tertiary hyperparathyroidism, atrioventricular conduction block, hemodialysis, end stage renal disease

## Abstract

The appearance of first-degree atrioventricular block and mitral annulus calcification in an end-stage renal failure patient with elevated parathyroid hormone levels should raise the suspicion of metastatic cardiac calcification. Measures should be taken to normalize the parathyroid hormone, calcium, and phosphorus levels to limit the progression of atrioventricular block. Exploration or removal of parathyroid glands should be considered if heart block worsens.

## Introduction

Since the End-Stage Renal Disease (ESRD) Program initiation in 1972, medical complications, including metastatic calcifications, have been rising [1,2]. Gradual deposition of calcium within the cardiac conduction system may predispose these patients to an increased incidence of arrhythmias, heart failure, and mortality. 

We present the case of a 68-year-old male previously diagnosed with ESRD on hemodialysis, who developed a third-degree atrioventricular (AV) block attributed to calcification of the mitral valve annulus. While this patient required placement of a permanent pacemaker, this report's objective was to retrospectively assess findings that may have predicted and allowed the ability to prevent this outcome.

## Case presentation

A 68-year-old male previously diagnosed with ESRD on hemodialysis presented to the ED due to lightheadedness and exertional dyspnea for the past several weeks. These symptoms were associated with multiple syncopal events that affected his quality of life. His past medical history was significant for hypertension and type 2 diabetes, and he was a social drinker but non-smoker with no adverse family history. His home medications included calcium acetate 667 mg thrice daily, cinacalcet 60 mg once daily, gabapentin 300 mg, rosuvastatin 40 mg, and thiamine 200 mg. 

**Figure 1:**
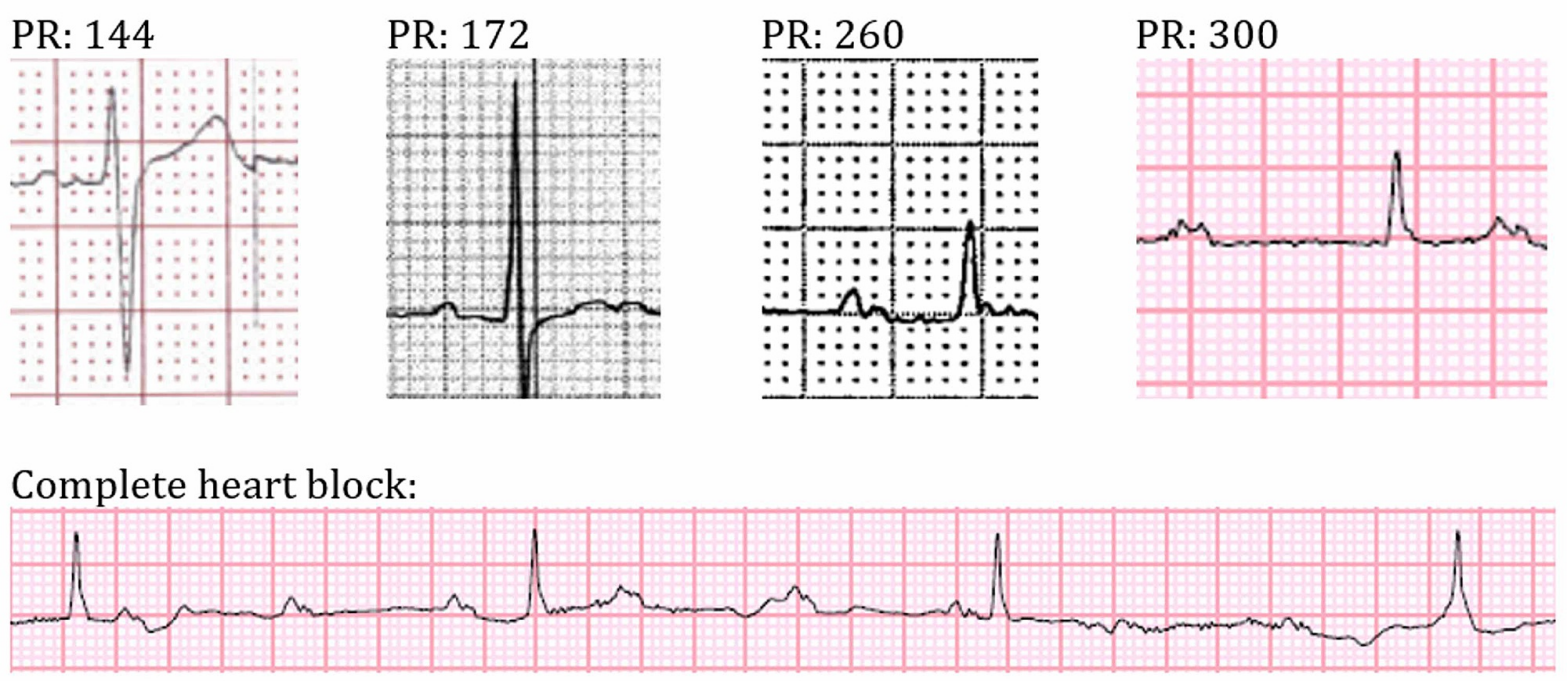
EKG illustrating sequential worsening of the atrioventricular block EKG: electrocardiogram

In the ED, his vital signs were stable, and he appeared to be in no distress. A cardiac examination was significant for 2 cm jugular venous distention and a mid-systolic click in the mitral area. While initial laboratory values, including calcium (Ca, corrected 9.6 mg/dL) and phosphorus (P, 3.7 mg/dL), were mostly unremarkable, parathyroid hormone (PTH) level was elevated at 737 pg/mL. An electrocardiogram (EKG ) in the ED revealed a first-degree AV block with a PR interval of 300 ms; however, telemetry was later notable for complete heart block (Figure 1). Electrophysiology placed an emergent temporary transvenous pacemaker, and he was admitted to the floor. The following day he received a dual-chamber permanent pacemaker. Subsequent transthoracic echocardiogram demonstrated severe mitral annular calcification (MAC; four videos in the Appendix), which was thought to be the underlying etiology for developing the cardiac conduction disorder.

**Figure 2:**
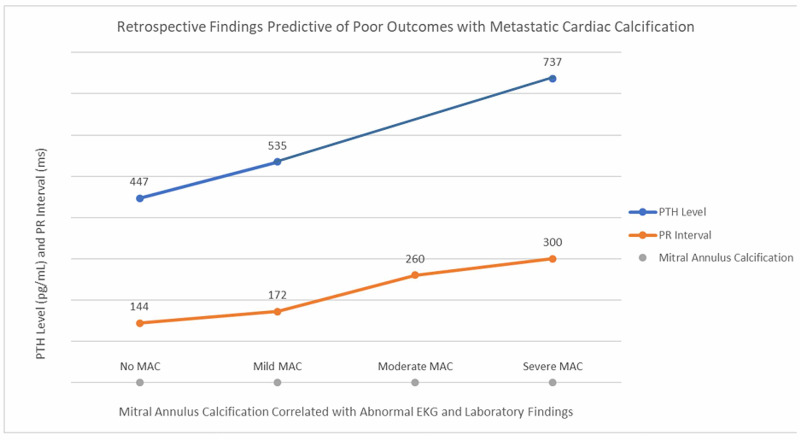
Shows worsening of PR interval and MAC as the serum PTH level increases MAC: mitral annulus calcification; PTH: parathyroid hormone

A retrospective chart review of prior serial testing demonstrated a progressively increasing PR interval and PTH level over the last several years. These findings appear to be directly correlated with worsening mitral annulus calcification (Table 1, Figure 2). Chest CT comparison between 2013 (without contrast) and 2019 (with contrast) shows worsening of MAC calcification from 2013 to 2019 (figure in the Appendix).

**Table 1:** Serial laboratory values, EKG, and echo findings Ca: calcium; Ca x P: calcium phosphate product; PTH: parathyroid hormone; EKG: electrocardiogram

MM/YY	Corrected Ca (mg/dL)	Ca x P	PR interval (ms)	Echo	PTH (pg/mL)
12/12	10.3	65	144	Normal study	447
09/14	11.1	71	172	Mild MAC	537
02/16	9.8	73	260	Moderate MAC	629
11/19	10	39	300	Severe MAC	737

## Discussion

Despite the prompt use of vitamin D metabolites in ESRD patients on hemodialysis, calcium and phosphorus disorders are common. These metabolic abnormalities can result in calcium deposition in normal healthy soft tissues, known as metastatic calcification. Metastatic calcification is an important etiology of cardiac conduction disorders for this subpopulation of patients.

Varma et al. demonstrated MAC to occur in 10-50% of ESRD patients [3]. Furthermore, Forman et al. demonstrated that MAC occurs more frequently in those receiving dialysis for an extended period [4]. This is notable for the current report as MAC is associated with a high frequency of conduction defects. Nair et al. found MAC in 87% of patients with symptomatic bradyarrhythmias [5]. While the association and mechanism between MAC and conduction disease have not been fully elucidated, the current hypothesis supports the notion that it may be partially due to the direct extension of calcium deposits in the AV node region and His bundle [6-8].

Proposed pathologic mechanisms in literature behind the metastatic calcifications in ESRD patients on hemodialysis include hyperparathyroidism, Ca and P products more than 72 influenced by dialysate calcium concentration, and vitamin D3 [9,10]. Few preliminary studies have shown that myocardial hypertrophy due to the elevated PTH and Ca have a similar effect. However, Barletta et al. hypothesized that PTH influences calcium entry into myocardial tissues independent of serum calcium level [11].

The salient features were metastatic calcification in the myocardium and the cardiac conduction system in a patient on cinacalcet and calcium acetate. Our patient was diagnosed with ESRD and secondary hyperparathyroidism about 10 years before this hospitalization and has been on dialysis since then. Serial echocardiogram obtained approximately every two to three years has shown a gradual increase in the MAC over the past 10 years. Serial EKG was also reviewed for the same duration and revealed a progressive PR interval prolongation that started after moderate MAC, which was seen on the transthoracic echocardiogram in 2017. Knowing that MAC is associated with an elevated calcium-phosphorus (Ca x P) product, hypercalcemia, and hyperphosphatemia - we reviewed his labs for the past 10 years that were available on the electronic health record. Records support a diagnosis of tertiary hyperparathyroidism with elevated PTH, elevated phosphorus even though he did not have overt hypercalcemia (Table 1). He was treated with calcimimetic drugs from early in the course of the disease process.

We conclude that the elevated PTH could be the driving force of calcium deposition in the mitral annulus in the absence of high calcium and elevated Ca x P. Prior case reports have demonstrated the reversal of AV block after parathyroidectomy [12,13]. However, there is a lack of evidence supporting early parathyroidectomy after first-degree AV block's appearance in long-term hemodialysis patients with secondary/tertiary hyperparathyroidism. Measures should be taken to optimize serum levels of PTH, calcium, and phosphate. Exploration and removal of parathyroid glands should be considered, when feasible, to prevent further progression of AV block.

## Conclusions

Long-term hemodialysis patients are predisposed to the development of cardiac conduction disorders. The appearance of a first-degree atrioventricular block in the setting of an elevated parathyroid hormone level and calcification of the mitral valve annulus may warrant serial EKG testing to assess for more pronounced deviations from the normal cardiac conduction. With the recognition of these findings, early consideration of parathyroidectomy should occur to prevent the worsening of the AV block. 

## Figures and Tables

**Figure 3:**
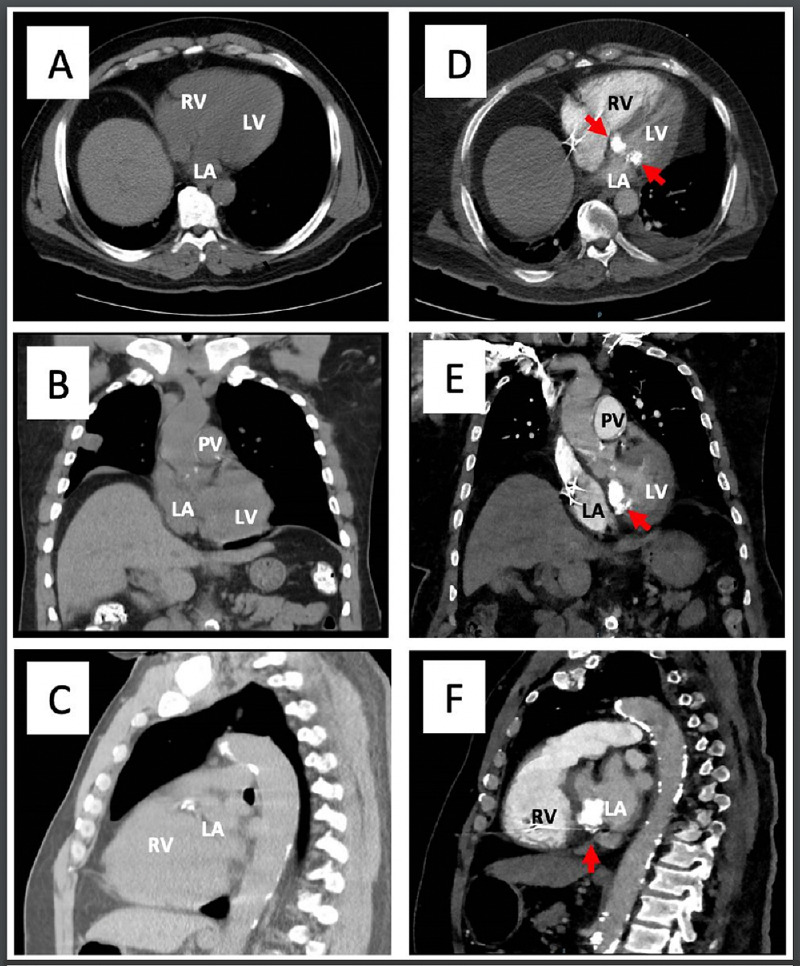
CT chest Chest CT comparison between 2013 (without contrast) and 2019 (with contrast). A. Axial view 2013; B. coronal view 2013; C. sagittal view 2013 D. axial view 2019; E. coronal view 2019; F. sagittal view 2019. Red arrow shows a heavily calcified mitral valve.

**Video 1:** Parasternal long-axis view

**Video 2:** Apical three-chamber view

**Video 3:** Apical four-chamber view

**Video 4:** Apical two-chamber view
